# Genetic Variation on 9p22 Is Associated with Abnormal Ovarian Ultrasound Results in the Prostate, Lung, Colorectal, and Ovarian Cancer Screening Trial

**DOI:** 10.1371/journal.pone.0021731

**Published:** 2011-07-07

**Authors:** Nicolas Wentzensen, Amanda Black, Kevin Jacobs, Hannah P. Yang, Christine D. Berg, Neil Caporaso, Ulrike Peters, Lawrence Ragard, Saundra S. Buys, Stephen Chanock, Patricia Hartge

**Affiliations:** 1 Division of Cancer Epidemiology and Genetics, National Cancer Institute, Rockville, Maryland, United States of America; 2 Core Genotyping Facility, National Cancer Institute, Rockville, Maryland, United States of America; 3 Division of Cancer Prevention, National Cancer Institute, Bethesda, Maryland, United States of America; 4 Cancer Prevention Program, Fred Hutchinson Cancer Research Center, Seattle, Washington, United States of America; 5 Westat, Rockville, Maryland, United States of America; 6 Oncology Division, Huntsman Cancer Institute, Salt Lake City, Utah, United States of America; Ohio State University Medical Center, United States of America

## Abstract

**Background:**

A recent ovarian cancer genome-wide association study (GWAS) identified a locus on 9p22 associated with reduced ovarian cancer risk. The single nucleotide polymorphism (SNP) markers localize to the *BNC2* gene, which has been associated with ovarian development.

**Methods:**

We analyzed the association of 9p22 SNPs with transvaginal ultrasound (TVU) screening results and CA-125 blood levels from participants without ovarian cancer in the Prostate, Lung, Colorectal, and Ovarian Cancer Screening Trial (PLCO); 1,106 women with adequate ultrasound screening results and available genotyping information were included in the study.

**Results:**

We observed a significantly increased risk of abnormal suspicious TVU results for seven SNPs on 9p22, with odds ratios between 1.68 (95% CI: 1.04–2.72) for rs4961501 and 2.10 (95% CI: 1.31–3.38) for rs12379183. Associations were restricted to abnormal suspicious findings at the first TVU screen. We did not observe an association between 9p22 SNPs and CA-125 levels.

**Conclusions:**

Our findings suggest that 9p22 SNPs, which were found to be associated with decreased risk of ovarian cancer in a recent GWAS, are associated with sonographically detectable ovarian abnormalities. Our results corroborate the relevance of the 9p22 locus for ovarian biology. Further studies are required to understand the complex relationship between screening abnormalities and ovarian carcinogenesis and to evaluate whether this locus can influence the risk stratification of ovarian cancer screening.

## Introduction

Ovarian cancer is the 8^th^ most common cancer and the 5^th^ leading cause of cancer death among women in the US [1]. Currently available early detection strategies are based on serum CA-125 measurement and transvaginal ultrasound (TVU) [2], [3]. However, these tests have not been shown to improve mortality from ovarian cancer; most women present at advanced disease stages [4].

The use of TVU is hampered by false-positive findings, resulting in unnecessary surgical procedures with possible complications [5],[6]. At the initial screening round in the Prostate, Lung, Colorectal, and Ovarian Cancer Screening Trial (PLCO), 1338 of 28,816 women (4.6%) were found to have abnormal TVU results, but only 13 invasive cancers were detected, translating to a positive predictive value of only 1% [2]. A recent evaluation of false positive TVU test results in PLCO showed that a wide variety of benign changes -not associated with ovarian cancer risk- are responsible for abnormal ultrasound findings [7]. A better understanding of ovarian cancer etiology is required to develop improved early detection and prevention strategies. Similarly, new approaches are needed to identify the subset of high risk women who might benefit from current screening modalities.

A recent genome wide association study identified the first ovarian cancer susceptibility locus with genome-wide significance [8]. A locus on 9p22 was associated with reduced ovarian cancer risk; the most significant SNP is rs3814113 (odds ratio = 0.82; p_trend_ 5.1×10^−19^). The locus includes the *basonuclein 2* (*BNC2*) gene; eight SNPs were located within intron 2 of the gene. *BNC2* is highly expressed in reproductive tissues and specifically implicated in oocyte development [9], [10]. Here, we analyzed the association of 9p22 SNPs with abnormal ovarian screening results among women in PLCO without ovarian cancer.

## Materials and Methods

We included all 1,106 women with TVU data and genome-wide scan data covering the 9p22 region from the PLCO screening arm (total n = 39,115 of whom n = 34,261 had not had prior oophorectomy). Each of the 10 screening centers obtained local Institutional Review Board approval to carry out the trial. NCI Institutional Review Board Approval was obtained to conduct genotyping among women who agreed to participate in genetic studies. We included only women with adequate TVU results and genotyping information for at least one of the SNPs on 9p22 previously found to be associated with ovarian cancer [8]. In total, 568 controls and 538 cases from genome wide association studies of pancreatic, lung, bladder, breast, renal, colon cancer, and glioma were included [11], [12]. Table 1 shows the distribution of cancer cases by site with the respective TVU results. Of note, most cancers developed during the follow-up of PLCO and were not present at the baseline TVU screen. TVU was performed at baseline and annually for four years according to the PLCO protocol at the screening centers [13]. TVU results were categorized as normal; abnormal not suspicious for ovarian cancer; and abnormal, suspicious for ovarian cancer as previously described [2]. Women with suspicious findings were referred for follow-up [14]. Ovaries were measured along the major and minor axes in both transverse and longitudinal planes, and the prolate ellipsoid formula (width×height×thickness×0.523) was used to calculate the volume of each ovary and/or cyst. In brief, the following findings were considered abnormal suspicious, i.e. screening positive: ovarian volume >10 cm^3^; cyst volume >10 cm^3^; any solid area or papillary projection extending into the cavity of a cystic ovarian tumor of any size; or any mixed (solid/cystic) component within a cystic ovarian tumor. CA-125 was measured using the Centocor CA-125II radioimmunoassay on serum prepared and frozen within 2 hours of blood draw [2].

**Table 1:** Risk factors and screening results in the subgroup with SNP data.

Variable	Baseline Abnormal TVU	Incident Abnormal TVU	Other TVU	Baseline vs. Incident/Otherx^2^ p-value	Baseline/Incident vs. Otherx^2^ p-value
**Age at entry**					
55–59	12 (24.0)	7 (15.2)	246 (24.4)		
60–64	15 (30.0)	13 (28.3)	321 (31.8)		
65–69	14 (28.0)	14 (30.4)	280 (27.7)		
70+	9 (18.0)	12 (26.1)	163 (16.1)	0.99	0.43
**Total**	**50**	**46**	**1010**		
**Race**					
Caucasian	43 (86.0)	37 (80.4)	912 (90.3)		
Non-Caucasian	7 (14.0)	9 (19.6)	98 (9.7)	0.38	0.03
**Total**	**50**	**46**	**1010**		
**Nulliparous**					
No	45 (90.0)	42 (91.3)	912 (90.4)		
Yes	5 (10.0)	4 (8.7)	97 (9.6)	0.92	0.94
**Total**	**50**	**46**	**1009**		
**Family hx breast cancer**					
No	44 (88.0)	36 (78.3)	850 (84.6)		
Yes, female relative	6 (12.0)	8 (17.4)	138 (13.7)		
Yes, male relative	0	1 (2.2)	2 (0.2)		
Possibly	0	1 (2.2)	15 (1.5)	0.78	0.48
**Total**	**50**	**46**	**1005**		
**Family hx ovarian cancer**					
No	47 (94.0)	41 (89.1)	949 (94.4)		
Yes, immediate family	3 (6.0)	3 (6.5)	40 (4.0)		
Possibly	0	2 (4.4)	16 (1.6)	0.53	0.53
**Total**	**50**	**46**	**1005**		
**PMH use**					
Ever	33 (66.0)	30 (65.2)	617 (61.1)		
Never	17 (34.0)	16 (34.8)	388 (38.5)		
Unknown	0	0	4 (0.4)	0.74	0.59
**Total**	**50**	**46**	**1009**		
**OC use**					
Never	23 (46.0)	26 (56.5)	511 (50.7)		
Ever	27 (54.0)	20 (43.5)	496 (49.3)	0.49	0.96
**Total**	**50**	**46**	**1007**		
**Smoker**					
Never	13 (26.0)	13 (28.3)	324 (32.1)		
Current	15 (30.0)	16 (34.8)	310 (30.7)		
Former	22 (44.0)	17 (37.0)	376 (37.2)	0.57	0.6
**Total**	**50**	**46**	**1010**		
**Benign cyst or tumor**					
No	35 (79.5)	37 (84.1)	867 (90.4)		
Yes	9 (20.5)	7 (15.9)	92 (9.6)	**0.02**	0.01
**Total**	**44**	**44**	**959**		
**Age of first menstrual period**					
<10	0	0	12 (1.2)		
10–11	8 (16.0)	12 (26.1)	167 (16.6)		
12–13	27 (54.0)	27 (58.7)	572 (56.8)		
14–15	12 (24.0)	5 (10.9)	215 (21.4)		
16+	3 (6.0)	2 (4.4)	41 (4.1)	0.86	0.57
**Total**	**50**	**46**	**1007**		
**GWAS case/control**					
Control	22 (44.0)	26 (56.5)	520 (51.5)		
Case	28 (56.0)	20 (43.5)	490 (48.5)	0.29	0.78
Bladder	4	6	84		
Breast	4	1	32		
Colon	4	1	79		
Lung	14	9	249		
Pancreas	2	3	46		
**Total**	**50**	**46**	**1010**		

TVU = transvaginal ultrasound; Family hx = family history; PMH = Post-menopausal hormone; OC = oral contraceptive; GWAS = genome-wide association study.

Genotyping was performed at the National Cancer Institute Core Genotyping Facility and Fred Hutchinson Cancer Research Center on the HumanHap550 Infinium II and Human 610-Quad chips (Illumina, San Diego, CA). Genotyping quality control followed standard procedures at the Core Genotyping Facility [11], [12]: DNA samples were selected for genotyping based on pre-genotyping quality control measures performed for the GWAS at the Core Genotyping Facility. Samples with less than 98% completion rate were excluded from the analysis and SNP assays with locus call rates lower than 90% were excluded. SNPs with extreme departures from Hardy-Weinberg proportions (*P*<1×10^−7^) were excluded from the primary analyses due to their increased likelihood of spurious associations due to problematic assays or genotyping calling. Ten 9p22 SNPs identified in the ovarian GWAS passed these quality control metrics and were included in the analysis: rs10756819, rs10810666, rs10962656, rs12379183, rs12379687, rs1339552, rs2153271, rs3814113, rs4961501, rs7861573.

**Figure 1:**
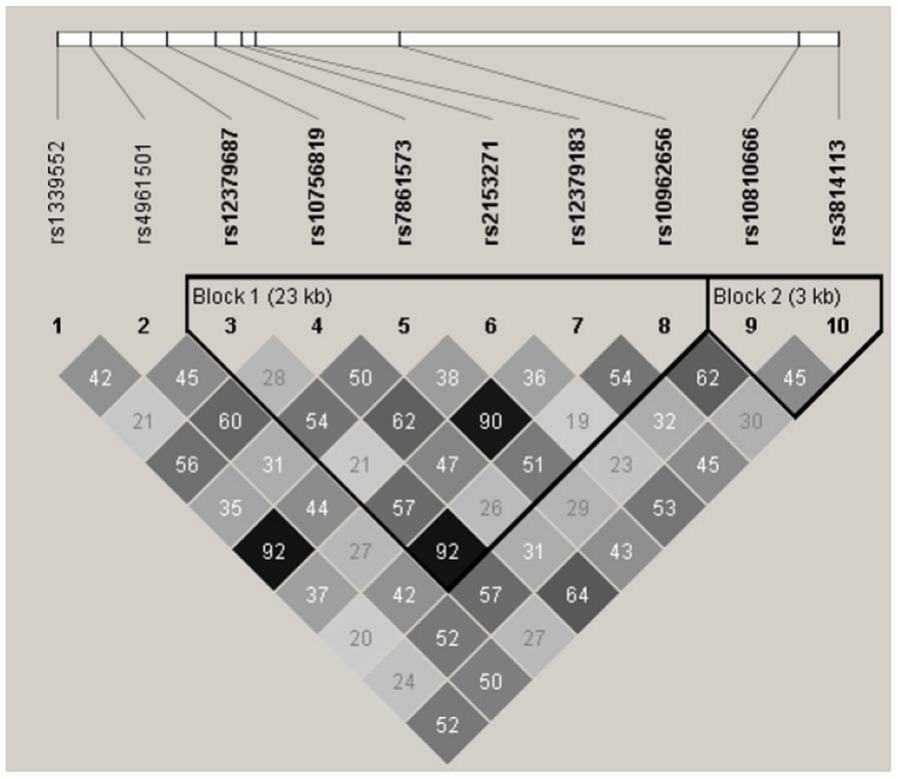
LD-plot of 10 SNPs on 9p22 from individuals included in the analysis. The LD-plot was generated with Haploview based on r^2^ of the 10 SNPs on 9p22 in 992 Caucasian women with genotyping information and transvaginal ultrasound results available.

TVU results were dichotomized into abnormal suspicious vs. normal and abnormal not suspicious. SNP associations were studied in relation to three outcomes: The TVU result at the first screen performed, the most severe TVU result of all screens, and the most severe incident TVU result (i.e. after excluding exams with abnormal screening results at the first screen). We used additive models defining the minor allele as risk allele to study the association of 9p22 SNPs with abnormal suspicious TVU results restricted to Caucasian individuals. We ran crude models and models adjusted for age as a continuous variable. For sensitivity analyses, we re-ran the models excluding individuals who were genotyped at the Fred Hutchinson Cancer Center but did not see any effect related to the site of genotyping. A sensitivity analysis restricted to control women only did not change the direction of the results. We used the Bonferroni correction as a conservative adjustment for multiple comparisons (n = 10), lowering the significance threshold to 0.005. Next, we studied the risk of abnormal TVU results associated with combinations of 0–2, 3–5, and 6–8 minor alleles from the four most significantly associated SNPs (rs10756819, rs12379183, rs3814113, and rs7861573). In addition, we combined the two least correlated SNPs (rs7861573 and rs10810666, Figure 1) and studied the association with abnormal TVU results creating three categories: Homozygous major alleles at both SNPs, one SNP with homozygous major alleles, and no SNP with homozygous major alleles. In all women, we analyzed the relationship between 9p22 SNPs and ovarian volume in 5-year age groups and by individual genotypes for rs12379183 and rs3814113. Further, among 43 Caucasian women with abnormal TVU results and genotyping data available, we explored the relationship between specific TVU characteristics including number of cysts, cyst diameter, and cyst volume with genotypes (dichotomized as AA vs. AB/BB) of rs10756819, rs12379183, and rs3814113.. In addition, we explored the association of 9p22 SNPs with CA-125 levels at baseline and with highest CA-125 levels measured at all screening visits stratifying by 5-year age groups over the complete age range of women included in this analysis (age at entry: 55–74 years). Haploview (http://www.broad.mit.edu/mpg/haploview/) was used to assess pair-wise linkage disequilibrium (LD) patterns among all women included in the analysis [15]. All statistical analyses were performed using SAS (SAS 9.1, SAS Institute, Cary, NC, USA).

## Results

Overall, 96 of the 1106 women included in this analysis had abnormal TVU screening results (8.7%) at any screening visit. In 50 women, abnormal TVU results were found at the first screening visit in PLCO (baseline abnormal TVU result), in the remaining 46 women, the abnormal TVU result was reported after an initially normal finding (incident abnormal TVU result).

Table 1 shows demographic and risk factor features of women included in this analysis, grouped in three categories: Abnormal TVU result at baseline, incident abnormal TVU results, and normal TVU results. Except for race and a previous history of benign cysts or tumors of the ovary, none of the demographic or risk factors summarized in Table 1 were associated with suspicious TVU results. All subsequent analyses were restricted to Caucasians only, leaving 43 women with baseline abnormal, 37 women with incident abnormal, and 912 women with non-suspicious TVU results.

Two SNPs, rs10756819 (OR = 1.48, p = 0.025) and rs12379183 (OR = 1.46, p = 0.046), showed a significant association with an abnormal TVU result at any time during follow up, while two other SNPs, rs3814113 and rs7861573, showed marginally significant results. None of these associations was significant after adjusting for multiple comparisons. After restricting to prevalent abnormal TVU findings as outcome only, the effect increased substantially: All ten SNPs on 9p22 showed increased ORs for suspicious TVU results at the first screen (Table 2) and seven SNPs showed significant ORs: rs12379183 (OR 2.10; p = 0.002), rs10756819 (OR 2.00; p = 0.002), rs7861573 (OR 1.99; p = 0.005), rs3814113 (OR 1.93; p = 0.005), rs10962656 (OR 1.73; p = 0.042), rs10810666 (OR 1.72; p = 0.031), and rs4961501 (OR 1.68; p = 0.04); the first four associations remained significant after adjusting for multiple comparisons. When restricting the TVU results to incident findings, none of the SNPs were associated with suspicious screening results. Adjusting for age did not change these results.

**Table 2:** Association of 10 SNPs on 9p22 with abnormal TVU screening results.

	Worst TVU result (n = 992)					First TVU result (n = 992)					Incident TVU result (n = 949)				
SNP	Case/Control	OR	Lower CL	Upper CL	P-value	Case/Control	OR	Lower CL	Upper CL	P-value	Case/Control	OR	Lower CL	Upper CL	P-value
**rs10756819**	80/912	1.48	1.05	2.08	**0.0254**	43/949	2.01	1.28	3.14	0.0024*	37/912	1.01	0.61	1.68	0.9763
**rs 10810666**	80/910	1.27	0.86	1.89	0.2275	43/947	1.72	1.05	2.82	**0.0307**	37/910	0.84	0.45	1.59	0.5982
**rs 10962656**	80/911	1.22	0.79	1.89	0.3684	43/948	1.73	1.02	2.94	**0.0423**	37/911	0.73	0.34	1.54	0.4024
**rs 12379183**	79/909	1.46	1.01	2.13	**0.0464**	42/946	2.10	1.31	3.38	0.0022*	37/909	0.90	0.50	1.64	0.7363
**rs 12379687**	79/911	1.21	0.78	1.87	0.3884	43/947	1.68	0.99	2.85	0.0527	36/911	0.74	0.35	1.55	0.4184
**rs 1339552**	75/790	1.10	0.78	1.56	0.5870	41/824	1.30	0.82	2.05	0.2602	34/790	0.90	0.54	1.50	0.6934
**rs 2153271**	78/911	1.12	0.80	1.58	0.5030	42/947	1.42	0.91	2.23	0.1241	36/911	0.85	0.51	1.41	0.5263
**rs 3814113**	79/912	1.39	0.98	1.97	0.0652	42/949	1.93	1.22	3.06	0.0049*	37/912	0.93	0.55	1.57	0.7849
**rs 4961501**	80/910	1.25	0.85	1.82	0.2563	43/947	1.68	1.04	2.72	**0.0352**	37/910	0.84	0.47	1.52	0.5711
**rs 7861573**	79/908	1.42	0.97	2.07	0.0694	42/945	1.99	1.23	3.21	0.0048*	37/908	0.90	0.50	1.64	0.7414

Per allele odds ratios obtained with an additive model restricted to the Caucasian population for the association of 9p22 SNPs with abnormal screening results are shown. Cases are women with suspicious screening results; controls are women with normal or non-suspicious screening results. Worst TVU results indicate abnormal TVU results at any screen during the 4-year follow-up. First TVU results indicate abnormal TVU results at the first screen a woman participated in. Incident TVU results are abnormal results among women that were normal or non-suspicious at the first screening. An asterisk indicates p-values lower than 0.005, the significance level after conservative Bonferroni correction.

Figure 1 shows an LD map of the 10 SNPs analyzed in the women from PLCO included in this analysis. Combinations of risk alleles from the four most significant SNPs showed significant associations with suspicious TVU results at the first screen (OR 1.95; p = 0.003). Women with combinations of the two least correlated significant SNPs (rs7861573 and rs10810666; r^2^ = 0.29; D′ = 0.63 in this population) had an OR of 1.61 (p = 0.007) for suspicious screening results (Table 3).

**Table 3:** Association of SNP combinations with abnormal TVU results.

SNP combination	Case/Control	OR	Lower CL	Upper CL	P-value
**rs10756819/rs12379183/rs3814113/rs7861573**	43/949	1.951	1.259	3.022	0.0028
**rs7861573/rs10810666**	43/949	1.616	1.141	2.290	0.0069

Per allele odds ratios obtained with an additive model restricted to the Caucasian population for the association of combinations of 9p22 SNPs with abnormal screening results are shown. First, combinations of the four most strongly associated SNPs were analyzed. Three groups were created based on the number of minor alleles: 0–2 alleles present, 3–5 alleles present, 6–8 alleles present. Next, the two least correlated SNPs were combined. For the two-SNP combination, homozygote major alleles were considered low risk, while heterozygous alleles and homozygous minor allele genotypes were considered high risk. Three groups were created as follows: low risk by both SNPs, high risk by either one of the SNPs, and high risk by both SNPs.

We explored the association of 9p22 SNPs with TVU characteristics and CA-125 levels. For the rs12379183 SNP that showed the strongest effect in this analysis, we observed a trend of increasing ovarian volume measured at first ultrasound associated with risk alleles in all age groups. For the rs3814113 SNP that was most strongly associated with ovarian cancer risk [8], increasing ovarian volume associated with risk alleles was only observed in the 55–59-year age group (Table 4). We analyzed the association of 9p22 genotypes with number, diameter, and volume of cysts among women with abnormal TVU results (Table 5). Interestingly, women carrying minor alleles had fewer cysts, with a mean number of 2.22 in women with two major alleles vs. 1.18 in women with at least one minor allele of rs10756819 (p = 0.01). In contrast, cyst volume was non-significantly higher in women carrying at least one minor allele. We did not observe an association between 9p22 SNPs and further TVU characteristics such as cyst volume and other cyst characteristics. To analyze whether 9p22 SNPs were associated with CA-125 levels, we compared the medians of the first and the maximal CA-125 values by genotype. None of the 10 SNPs studied showed an association with CA-125 levels (data not shown).

**Table 4:** 9p22 genotypes and ovarian volume.

SNP	Genotype	Age	N	First volume median cm^3^ (IQR)	Maximal volume median cm^3^ (IQR)
**rs12379183**	AA	**All**	**518**	**1.2 (1.6)**	**1.6 (2.0)**
		55–59	122	1.2 (1.6)	1.6 (2.3)
		60–64	169	1.2 (1.8)	1.6 (2.3)
		65–69	141	1.2 (1.3)	1.6 (1.6)
		70–74	86	1.1 (1.6)	1.65 (2.4)
	AG	**All**	**291**	**1.3 (1.6)**	**1.8 (2.1)**
		55–59	71	1.5 (1.7)	1.8 (1.4)
		60–64	93	1.4 (2.2)	2.1 (2.7)
		65–69	77	1.4 (1.6	1.6 (2.1)
		70–74	50	1 (1.2)	1.35 (1.3)
	GG	**All**	**36**	**1.6 (1.45)**	**1.9 (2.95)**
		55–59	8	1.95 (1.2)	1.95 (1.85)
		60–64	12	1.7 (1.35)	2.25 (3.85)
		65–69	11	1.6 (1.7)	2.4 (3.0)
		70–74	5	1.2 (0.6)	1.4 (0.4)
**rs3814113**	TT	**All**	**393**	**1.2 (1.5)**	**1.6 (1.8)**
		55–59	97	1.3 (1.8)	1.7 (2.3)
		60–64	125	1.2 (1.7)	1.6 (2.1)
		65–69	109	1.2 (1.2)	1.5 (1.4)
		70–74	62	1.1 (1.7)	1.45 (2.1)
	TC	**All**	**379**	**1.3 (1.8)**	**1.8 (2.1)**
		55–59	94	1.4 (1.6)	1.7 (1.6)
		60–64	125	1.4 (2.2)	2.1 (2.9)
		65–69	92	1.4 (1.65)	1.6 (1.8)
		70–74	68	1.1 (1.25)	1.7 (1.95)
	CC	**All**	**76**	**1.2 (1.5)**	**1.8 (2.4)**
		55–59	11	2 (1.8)	2.1 (3.1)
		60–64	26	1.2 (1)	1.85 (2.2)
		65–69	29	1.3 (1.7)	2.1 (3.1)
		70–74	10	0.9 (0.5)	1.05 (0.7)

Median ovarian volume and interquartile range at the first visit and median of the highest measured volume per woman is shown stratified by genotypes and age groups.

**Table 5:** 9p22 SNPs and ovarian cyst characteristics in TVU.

		RS12379183			RS10756819			RS3814113		
		AA (n = 16)	AB/BB (n = 25)	p-value	AA (n = 10)	AB/BB (n = 32)	p-value	AA (n = 11)	AB/BB (n = 30)	p-value
Number of cysts	Mean	1.69	1.23	0.21	2.22	1.18	**0.01**	1.82	1.19	0.08
	SE	0.38	0.15		0.64	0.12		0.46	0.13	
Cyst diameter (cm)	Mean	3.87	3.74	0.81	3.56	3.86	0.48	3.83	3.76	0.92
	SE	0.24	0.46		0.36	0.36		0.39	0.38	
Cyst volume (cm^3^)	Mean	35.38	60.78	0.21	29.36	56.92	0.07	38.03	55.5	0.34
	SE	5.3	18.85		7.46	14.79		9.14	15.72	

A = major allele; B = minor allele. SE = standard error. T-test p-values are shown.

## Discussion

A large consortial GWAS effort recently identified several SNPs on 9p22 that are associated with ovarian cancer risk [8]. The SNPs are located in the region of the *BNC2* gene which is involved in ovarian development [9], [10]. Spurred by these independent prior findings on 9p22/*BNC2* and ovarian biology we sought to leverage available TVU and genetic data to study the association between genetic variation and abnormal ovarian ultrasound findings. Our study is an example of an exploration of biological mechanisms following GWAS. Ovarian cancer screening using ultrasound and CA-125 testing is currently evaluated in two large randomized trials in the US and the UK. Previous analyses in PLCO have shown that the positive predictive value of TVU-based screening is low; almost all women with abnormal ultrasound findings do not have and do not develop ovarian cancer [2]. Therefore, we do consider these TVU findings as surrogates for biological changes occurring in the ovary (with carcinogenic changes being one option), rather than surrogates for cancer.

In our study of women without ovarian cancer, we observed a significantly increased risk of abnormal suspicious TVU results for several SNPs on 9p22 that have been found to be associated with reduced ovarian cancer risk [8]. We did not expect that SNPs associated with reduced ovarian cancer risk would correlate positively with abnormalities on ultrasound. Although the findings appear perplexing at first sight, it is conceivable that SNPs found to lower the risk of ovarian cancer may be associated with prevalent abnormal TVU findings.

We explored the association of 9p22 genotypes with morphologic characteristics recorded during TVU in women with abnormal TVU screening results. Although numbers were limited, we observed that women carrying minor 9p22 alleles had ultrasound features corresponding to complex ovarian cysts [16]. In a previous analysis in PLCO, women with complex cysts were not found to share established risk factors for ovarian malignancy [16]. In a more recent analysis in PLCO, the risk of ovarian cancer among women with prevalent cysts was slightly, but non-significantly lower compared to women with no cysts [17]. Unfortunately, histology reports of benign outcomes in women treated for abnormal TVU results were not systematically collected in PLCO and could not be evaluated in relation to 9p22 genotypes.

Most importantly, our findings require independent confirmation, which is challenging, as there are only few resources that provide both TVU screening information and genetic data from a population-based study. If confirmed, our data suggest that some genes potentially protective against ovarian cancer actually are associated with suspicious TVU findings such as increased ovarian volume or complex cysts that gradually arise over decades and are detected at the first TVU screen.

The biology of ovarian cancer development is not well understood. It has been suggested that incessant ovulation, associated with repeated disruption and micro-traumas of the ovarian surface epithelium, may lead to initial transformation [18]. Other theories suggest that hormonal stimulation of the epithelium, especially by estrogens and estrogen metabolites, may initiate carcinogenesis [19]. There is now growing evidence that at least a subset of ovarian cancers may arise in the Fallopian tube and implant in the ovaries early on [20].

Ovarian abnormalities associated with SNPs at the 9p22 locus may protect against cancer development by interfering with these carcinogenic mechanisms, e.g. by reducing the number of lifetime ovulations or by modulating the exposure of ovarian tissue to endogenous or exogenous hormones. Ovarian cysts may impede implantation of early transformed cell clones derived from the Fallopian tube. Furthermore, although we did not see any evidence in PLCO, we cannot exclude that the reduced ovarian cancer risk associated with these SNPs is related to more frequent oophorectomies following suspicious TVU results, rather than to a direct biological mechanism.

If the 9p22 locus is associated with false positive ovarian cancer screening results, genotyping might have influence on the interpretation of TVU results.

A recent study demonstrated that cancer-related SNPs may influence prostate cancer risk estimates related to prostate specific antigen levels [21]. In a study of breast cancer risk models, 10 common genetic variants associated with breast cancer risk had similar performance as the Gail model based on clinical at predicting breast cancer risk, but adding the SNPs to the clinical data only modestly improved risk prediction [22]. Replication of our findings in other studies, evaluation of risk factors associated with the 9p22 locus and extension to ovarian cancer cases are necessary to understand the complex relationship between screening abnormalities and ovarian carcinogenesis and to evaluate whether this locus can influence the risk stratification of TVU screening. Moreover, detailed mapping of the region is needed to identify the actual ‘at risk’ and protective haplotypes.
